# RabGDI controls axonal midline crossing by regulating Robo1 surface expression

**DOI:** 10.1186/1749-8104-7-36

**Published:** 2012-11-09

**Authors:** Melanie Philipp, Vera Niederkofler, Marc Debrunner, Tobias Alther, Beat Kunz, Esther T Stoeckli

**Affiliations:** 1 Institute for Biochemistry and Molecular Biology, University of Ulm, Ulm, Germany; 2 Department of Genetics, Harvard Medical School, Boston, USA; 3 Institute of Molecular Life Sciences, University of Zurich, Winterthurerstrasse 190, Zurich, CH, 8057, Switzerland

**Keywords:** Axon guidance, In ovo RNAi, Slit, Membrane trafficking, Chicken embryo, Spinal cord development, Mental retardation gene

## Abstract

**Background:**

Axons navigate to their future synaptic targets with the help of choice points, intermediate targets that express axon guidance cues. Once they reach a choice point, axons need to switch their response from attraction to repulsion in order to move on with the next stage of their journey. The mechanisms underlying the change in axonal responsiveness are poorly understood. Commissural axons become sensitive to the repulsive activity of Slits when they cross the ventral midline of the CNS. Responsiveness to Slits depends on surface expression of Robo receptors. In *Drosophila*, Commissureless (Comm) plays a crucial regulatory role in midline crossing by keeping Robo levels low on precommissural axons. Interestingly, to date no vertebrate homolog of *comm* has been identified. Robo3/Rig1 has been shown to control Slit sensitivity before the midline, but without affecting Robo1 surface expression.

**Results:**

We had identified *RabGDI*, a gene linked to human mental retardation and an essential component of the vesicle fusion machinery, in a screen for differentially expressed floor-plate genes. Downregulation of RabGDI by in ovo RNAi caused commissural axons to stall in the floor plate, phenocopying the effect observed after downregulation of Robo1. Conversely, premature expression of RabGDI prevented commissural axons from entering the floor plate. Furthermore, RabGDI triggered Robo1 surface expression in cultured commissural neurons. Taken together, our results identify RabGDI as a component of the switching mechanism that is required for commissural axons to change their response from attraction to repulsion at the intermediate target.

**Conclusion:**

RabGDI takes over the functional role of fly Comm by regulating the surface expression of Robo1 on commissural axons in vertebrates. This in turn allows commissural axons to switch from attraction to repulsion at the midline of the spinal cord.

## Background

The current model of axon guidance postulates a collaboration of attractive and repulsive guidance cues that can act over some distance as long-range guidance cues, or locally as short-range guidance cues [1,2]. One of the preferred systems for axon guidance studies has been commissural axons that cross the floor plate, the ventral midline of the spinal cord [3,4]. Midline crossing is a conserved feature of axonal navigation between invertebrates and vertebrates [4-6]. In vertebrates, axons are guided toward the ventral midline by long-range guidance cues. These include roof plate-derived BMPs and Draxin which repel commissural axons from the dorsal midline [7-9] and the chemoattractants Netrin-1 and Shh which are released from the floor plate [10]. In both, the ventral nerve cord of invertebrates and the vertebrate spinal cord, midline crossing is controlled by a balance between positive and negative signals derived from the interaction between growth cone receptors and ligands expressed by midline cells [3,11]. Negative regulators of midline crossing were first identified based on genetic screens in *Drosophila*[12,13]. Characterization of the genes responsible for this repulsive activity identified *robo* receptors [14] (*roundabout* receptors) and their ligand Slit [15] but also the transmembrane protein *comm* (*commissureless*), which was found to regulate Robo expression [16-19]. In vertebrates, positive regulators of midline crossing were first identified [20]. Both in vivo and in vitro interactions of Axonin-1/TAG-1/Contactin-2 and NrCAM were shown to mask a repellent activity of the floor plate [21,22]. The repellent activity was later attributed to Semaphorin 3B and 3F, mediated by Neuropilin-2 [23], and to orthologs of *Drosophila* Slit, mediated by Robo receptors [15,23-29]. Vertebrates express three *Slits*[25,30-33] and four *Robos*: *Robo1*, *Robo2*, and *Robo3/Rig1* are expressed in the developing nervous system [34,35]. Robo4 (Magic Roundabout) differs markedly in its domain structure from the other Robos and is expressed exclusively in endothelial cells [36,37]. A role for Robo4 in angiogenesis has been described in mice [38] and zebrafish [39].

In the developing nervous system, Robos were mainly described as receptors for Slits which mediate a repellent signal. For midline crossing, commissural neurons face the problem of regulating Robo expression temporally in such a way that Robo is not expressed on the axonal surface before they have reached and entered the floor plate. However, upon floor-plate contact Robo has to be expressed on commissural growth cones in order to expel them from the floor plate that was previously perceived as an attractive environment.

The model of Robo regulation put forth in invertebrates postulates that midline crossing is controlled by Comm, which prevents surface expression of Robo before midline contact [16,17,40-44]. According to the sorting model, *comm* is specifically and transiently expressed in contralaterally but not ipsilaterally projecting neurons. In the presence of Comm, Robo is not inserted into the plasma membrane but rather transported to the endosomal-lysosomal compartment directly, thus allowing axons to cross the midline [18,19].

Interestingly, an ortholog of *comm* is not found in vertebrate genomes [41,43], and therefore, it has been unclear how Robo levels are controlled in vertebrate commissural axons. A role for Robo3/Rig-1 in regulating the function of Robo1 as receptor for midline Slits has been suggested, but the proposed mechanism does not include the regulation of Robo1 levels on precommissural axons [28]. Instead, alternative splicing was recently reported to produce different Robo3 isoforms with antagonistic function with respect to midline crossing [45]. Robo3.1 was shown to be expressed on axons before, whereas Robo3.2 is expressed after midline crossing. Based on loss- and gain-of-function experiments, the authors suggested that Robo3.1 silences the effect of Robo1 and Robo2, while Robo3.2 enhances their effect and perhaps additionally counteracts Robo3.1 function. Still, it remains unclear how Robo1 protein levels are kept low on pre-crossing compared to post-crossing axons, a finding that was confirmed in several studies.

Here, we show that levels of Robo1 on commissural axons are regulated by RabGDI (Rab Guanine Nucleotide Dissociation Inhibitor, GDI1). RabGDI is a component of the vesicle fusion machinery [46,47]. It is required for the recycling of hydrolyzed RabGDP to RabGTP. RabGDI retrieves RabGDP from the plasma membrane and shuttles it to new donor vesicles, where RabGDP is activated by a guanine nucleotide exchange factor (GEF). The GEF exchanges the GDP for a GTP, thus recycling the active RabGTP required for a subsequent round of vesicle fusion.

In humans, loss of *RabGDI* function results in mental retardation [48]. In mice, loss of *RabGDI* function has been associated with defects in associative memory [49]. These abnormalities are linked to changes in Rab-mediated vesicle trafficking. Here, we provide in vivo and in vitro evidence that loss of *RabGDI* function during midline crossing prevents the fusion of a subset of vesicles required for the insertion of Robo1 into the growth cone membrane. Thus, in both invertebrates and vertebrates, Robo levels on precommissural axons are regulated post-translationally to allow midline crossing. However, the mechanisms and the molecules involved in the regulation of Robo1 surface levels differ: In flies commissural axons can cross the midline, because the transient expression of Comm prevents Robo1 surface expression by directing it to the lysosomal pathway. In chicken, RabGDI is required for membrane-insertion of Robo1. In the absence of RabGDI, Robo1 is not inserted into the growth cone surface.

## Results

### RabGDI is required for commissural axons to cross the floor plate in the embryonic chicken spinal cord

**Figure 1:**
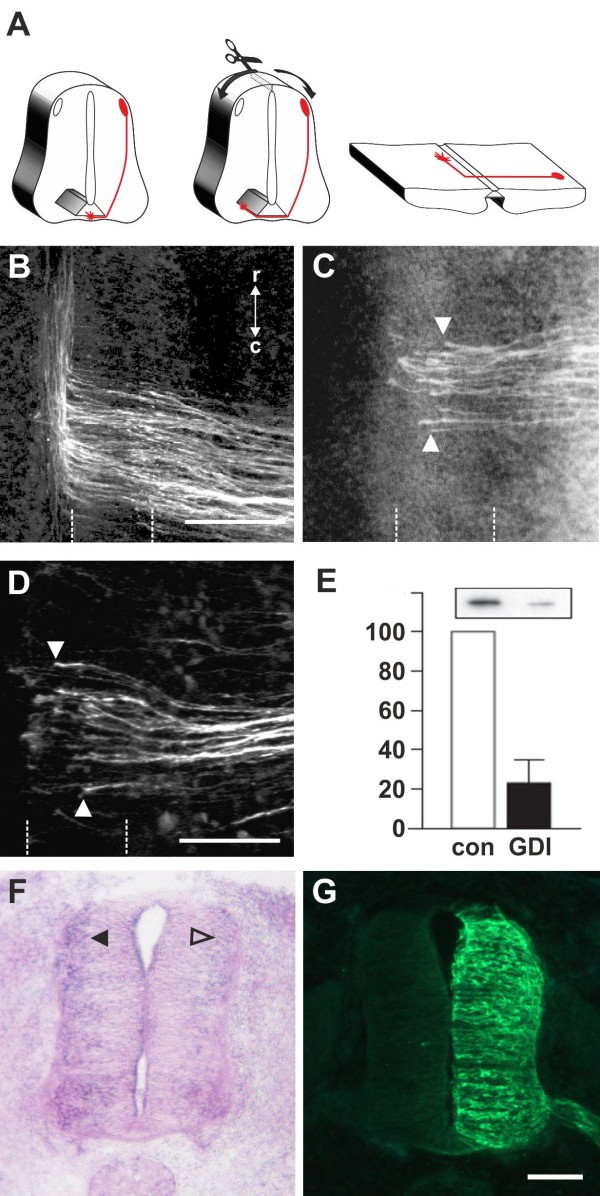
**Commissural axons fail to reach the contralateral floor-plate border in the absence of RabGDI.** Dorsolateral commissural neurons (dI1; shown in red) extend their axons ventromedially toward the floor plate, where they cross the midline. After reaching the contralateral floor-plate border axons turn rostrally into the longitudinal axis, in close contact with the floor-plate border (**A**). Commissural axon pathfinding was visualized in ‘open-book’ preparations by injecting the lipophilic dye DiI into the area of their cell bodies. At HH25, commissural axons in control-injected (**B**) and in non-injected embryos (not shown) had crossed the floor plate and extended a considerable distance along the longitudinal axis of the spinal cord. In the absence of RabGDI, commissural axons entered the floor plate (indicated by the dashed lines) but most of them failed to reach the contralateral floor-plate border (arrowheads in **C**) in age-matched embryos. The same phenotypes were obtained by injection of dsRNA derived from independent, non-overlapping cDNA fragments from the coding region (**C**) and the 3’ UTR (**D**). The downregulation of RabGDI was quantified in HEK293 cells using Western blots. RNAi reduced RabGDI protein levels by 77 ± 12%, compared to controls (**E**). Efficient downregulation of *RabGDI* mRNA by *in ovo* RNAi on the electroporated (right side) compared to the control (left) side of the spinal cord could also be observed by in situ hybridization at HH23/24 (**F**). A vector encoding YFP was co-injected to verify efficient transfection (**G**). Bar 50 μm. Rostral is to the top in **B** - **D** .

We identified *RabGDI* in a subtractive hybridization screen for guidance cues affecting navigation of commissural axons at the midline of the chicken spinal cord [50,51]. After downregulation of RabGDI by in ovo RNAi using long double-stranded RNA (dsRNA), commissural axons stalled within the floor plate and failed to reach the contralateral border (Figure 1; see also [50]). Growth toward and into the floor plate was not affected (compare Figure 1BB and C). The same results were obtained with dsRNA derived from a second, non-overlapping cDNA fragment from the 3’ UTR of RabGDI (Figure 1D). Silencing *RabGDI* by RNAi in HEK cells reduced protein levels by 77% (Figure 1E). Similarly, a strong reduction of the mRNA level was seen in spinal cord sections subject to in situ hybridization (Figure 1F) after one-sided electroporation of the neural tube (Figure 1G).

Downregulation of RabGDI by electroporation of only the dorsal spinal cord did not change the observed phenotype, indicating that RabGDI is required cell autonomously in commissural neurons for correct midline crossing of their axons. After electroporation of only the dorsal spinal cord aberrant midline crossing of commissural axons was observed at 72.3% of the injection sites compared to 70.6% of the injection sites when one side of the spinal cord was electroporated (Additional file 1: Figure S1). The effect on commissural axon guidance was direct and not a consequence of aberrant patterning of the spinal cord, as we did not observe any changes in the expression of Pax3, Nkx2.2, or Isl1 (Additional file 2: Figure S2).

Evidence for the specificity of the effect was obtained in a rescue experiment. The stalling phenotype obtained with dsRNA derived from the 3’ UTR could be partially rescued by co-electroporation of a plasmid containing the open-reading frame of RabGDI. Under these conditions the stalling phenotype was reduced by 43.8% (n = 170 injection sites in 16 embryos for dsRabGDI + RabGDI and n = 140 injection sites in a total of 14 embryos for dsRabGDI).

The stalling phenotype observed after downregulation of RabGDI could be explained by a failure in the shift from positive to negative guidance signals, which is required for successful midline crossing. According to this shift-of-balance model, RabGDI would be necessary for the fusion of a subtype of vesicles containing specific axon guidance receptors that allow for a switch in pathfinding behavior at choice points, such as the floor plate [3,4]. According to this model, axons would still be able to extend into the floor plate in the absence of RabGDI, but they would not leave the floor plate area, because they fail to sense negative cues associated with the floor plate that change their responsiveness and drive them out of the floor plate.

### Decreasing positive cues can counteract *RabGDI* loss of function

**Figure 2:**
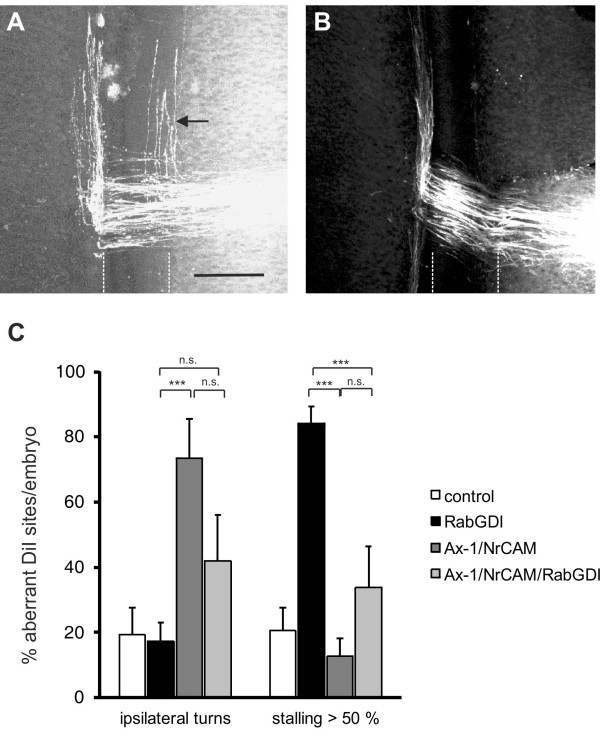
**RabGDI is required for the detection of negative cues at the midline.** In the absence of Axonin-1/NrCAM interactions, some axons failed to enter the floor plate (indicated by dashed lines) and turned prematurely into the longitudinal axis along the ipsilateral floor-plate border (arrow in **A**). This Axonin-1/NrCAM phenotype was partially rescued when RabGDI was downregulated together with Axonin-1 and NrCAM (**B**,**C**). Similarly, the concomitant silencing of *Axonin*-*1/NrCAM* resulted in a partial rescue of the axon stalling phenotype observed following downregulation of RabGDI alone (**C**). Quantification of the phenotypes (see Methods) is shown in (**C**). n.s. = not significant, * p<0.05, ** p<0.01, *** p<0.001. Graphs show mean + SEM in each case. Bar: 50 μm.

To test whether the failure of commissural axons to cross and exit the floor plate in the absence of RabGDI function was indeed due to the lack of the shift from positive to negative signals, we manipulated the balance experimentally. Axonin-1/Contactin-2 on commissural axons and NrCAM on floor-plate cells were shown previously to be required for midline crossing, because they provide positive cues that allow precommissural axons to enter the floor plate [20,21,50]. Thus, we downregulated Axonin-1 and NrCAM to disrupt positive axon guidance cues, and then assessed the incidence of ipsilaterally turning axons following the concomitant downregulation of RabGDI (Figure 2). Loss of Axonin-1 and NrCAM after in ovo RNAi resulted in the failure of commissural axons to cross the midline as shown previously [50]. Erroneous ipsilateral turns were observed at 73.6 ± 12.1% of the injection sites (n = 49, 9 embryos; Figure 2A,C). When RabGDI was downregulated concomitantly, ipsilateral turns (i.e. axons failing to enter the floor plate), were seen at only 42.0 ± 14.0% of the injection sites (n = 35, 8 embryos; Figure 2C). Thus, the concomitant silencing of *RabGDI* increased the likelihood of axons entering and crossing the floor plate following downregulation of positive cues (Figure 2B). This finding suggested that the lack of RabGDI was able to counteract guidance errors resulting from the reduction of positive cues, and was consistent with RabGDI acting as a negative regulator of midline crossing. In support of this balance model, axons did not fail to *enter* the floor plate in the absence of negative cues, but rather failed to *leave*. Thus, the percentage of injection sites with ipsilaterally turning axons after silencing *RabGDI* alone was not different from control (17.2 ± 5.8% and 19.3 ± 8.3%, respectively).

The balance model was further supported when the *RabGDI* phenotype was quantified. Following the loss of RabGDI, that is when there is an excess of positive stimuli, axons stalled and failed to reach the contralateral floor-plate border at 84.2 ± 5.1% of the injection sites (n = 118; 17 embryos; Figure 1C and Figure 2C). As expected, concomitant downregulation of Axonin-1, NrCAM, and RabGDI rescued the stalling phenotype. Knocking down all three genes resulted at a stalling phenotype in only 33.7 ± 12.6% of the injection sites (n = 35; 8 embryos). Taken together, these results are consistent with the idea that the lack of positive stimuli can be counteracted by a decrease in negative stimuli.

### Loss of *Slit* function mimics the *RabGDI* loss-of-function phenotype

For the identification of the negative cues that might play a role in counteracting Axonin-1/NrCAM interactions in a RabGDI-dependent manner, we focused on Slits rather than Semaphorins. In mouse, Neuropilin-2, a receptor component for Semaphorins, has been shown to expel commissural axons from the floor plate [23]. However, in chick Neuropilin-2 is not prominently expressed in dorsolateral commissural neurons when their axons are crossing the floor plate [52].

**Figure 3:**
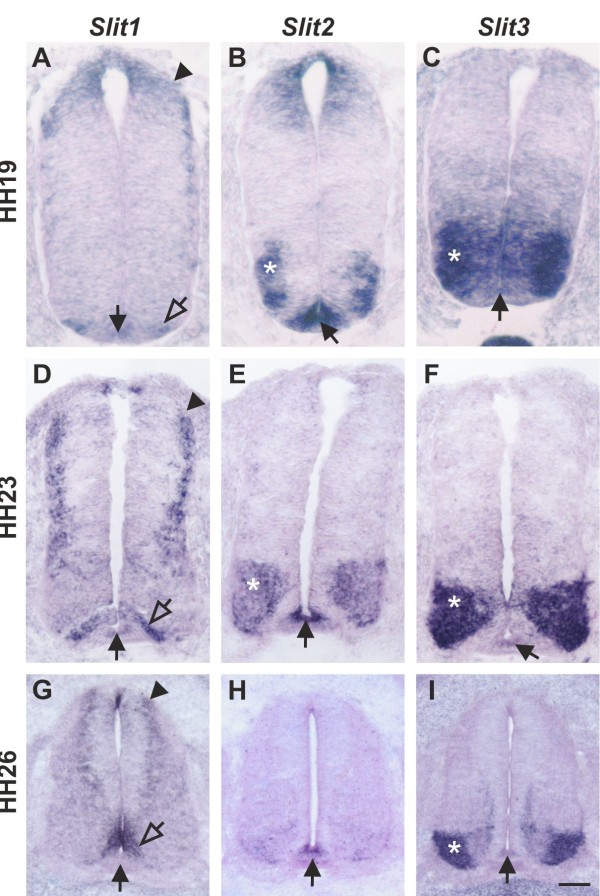
***Slits* are expressed in the floor-plate area of the embryonic chicken spinal cord.** In agreement with published reports [29,68], *Slit1* was expressed weakly in the floor plate at HH19, when commissural axons start to extend in the dorsal spinal cord but before they reach the floor plate (black arrow in **A**). Interestingly, *Slit1* was also expressed in commissural neurons throughout axonal pathfinding (HH19-26; arrowheads in **ADG**). *Slit1* was expressed more strongly in cells flanking the floor plate (open arrows in **ADG**) rather than the floor plate itself (arrow; **DG**). By HH26, *Slit1* was no longer expressed in the floor plate (arrow in **G**) but persisted in cells flanking the floor plate. *Slit2* was strongly expressed in the floor plate (arrow in **BEH**), in agreement with published findings [29,68]. Furthermore, *Slit2* was expressed by motoneurons (asterisks in **BE**). *Slit3* was strongly expressed in motoneurons (asterisks in **CFI**), but only at low levels, if at all, in the floor plate (arrows in **CFI**). Thus *Slit* expression shows some differences between chicken and rodent embryos [25,33]. In mouse [33] and in rat [25], *Slit1* is expressed in the floor plate rather than in cells flanking the floor plate. Mouse but not rat motoneurons express *Slit3* between E10.5 to E13.5. Motoneurons are the predominant site of *Slit3* expression in the chick at HH23. Overall, *Slit* expression is conserved between chicken embryos and rodents, although the patterns of individual *Slits* differ significantly. Bar: 33 μm in **AC**, 50 μm in **DF**, 100 μm in **GI**.

Slits have been described as negative regulators of midline crossing in both invertebrates [15,53] and vertebrates [25,27,28,54,55]. Based on our expression analysis, Slit1 and Slit2 made good candidates as midline repellents in the chicken embryo (Figure 3).

**Figure 4:**
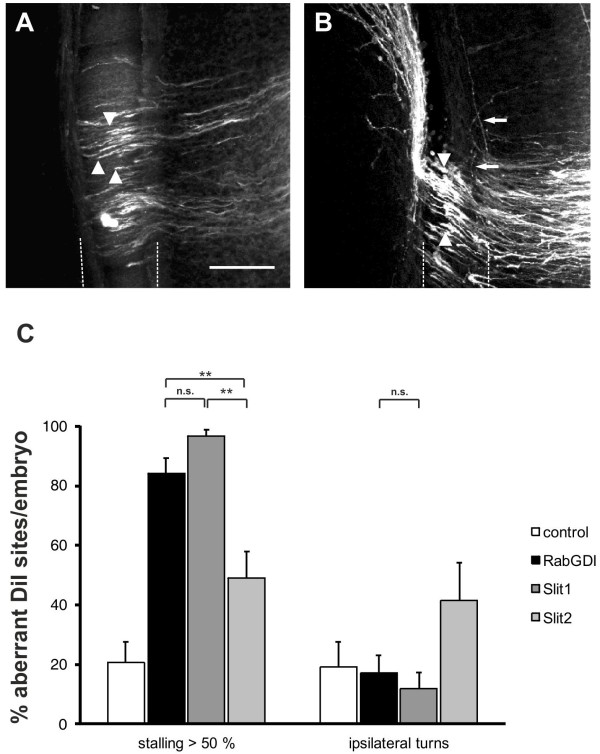
**The reduction of negative cues associated with the floor plate by downregulation of Slit1 reproduces the RabGDI phenotype.** Silencing *Slit1* (**A**) or *Slit2* (**B**) by in ovo RNAi resulted in stalling of commissural axons (arrowheads) in the floor plate (dashed lines), similar to the phenotype observed after loss of *RabGDI* function (compare to Figure 1C and D). However, the effect of loss of Slit2 on commissural axon stalling was significantly weaker than the effect of Slit1 downregulation (**C**). Loss of *Slit1* or *Slit2* function also revealed qualitative phenotypic differences between the two. The *Slit1* phenotype was the same as the one seen in the absence of RabGDI (compare **A** with Figure 1C, D). In contrast, loss of Slit2 additionally produced axons that turned into the longitudinal axis prematurely (arrows in **B**), an abnormality that was only rarely seen after downregulation of Slit1 (**C**). Phenotype quantifications are shown in (**C**). Significance levels: n.s. not significant, ** p<0.01. Values for control and RabGDI taken from Figure 2C. Bar: 50 μm.

If our model is correct, then loss of *Slit* function, i.e. the negative cues associated with the floor-plate area, should be equivalent to the loss of *RabGDI* function. Indeed, this is what we found by downregulating *Slits* by in ovo RNAi (Figure 4). Loss of *Slit1* resulted in a stalling phenotype at almost all injection sites (96.7 ± 2.1%; n = 40, 6 embryos; Figure 4A,C). The effect of *Slit2* downregulation was less pronounced, resulting in a stalling phenotype at 49.1 ± 8.9% of the injection sites (n = 91, 12 embryos). Interestingly, there was also a qualitative difference between *Slit1* and *Slit2* downregulation. In the absence of Slit2 some axons were found to turn ipsilaterally at 41.6 ± 12.7% of the injection sites. In contrast, ipsilateral turns were found only at 11.8 ± 5.4% of the injection sites of embryos lacking Slit1.

### *Robo* expression in dorsolateral commissural neurons is the same in chicken and rodent spinal cord

**Figure 5:**
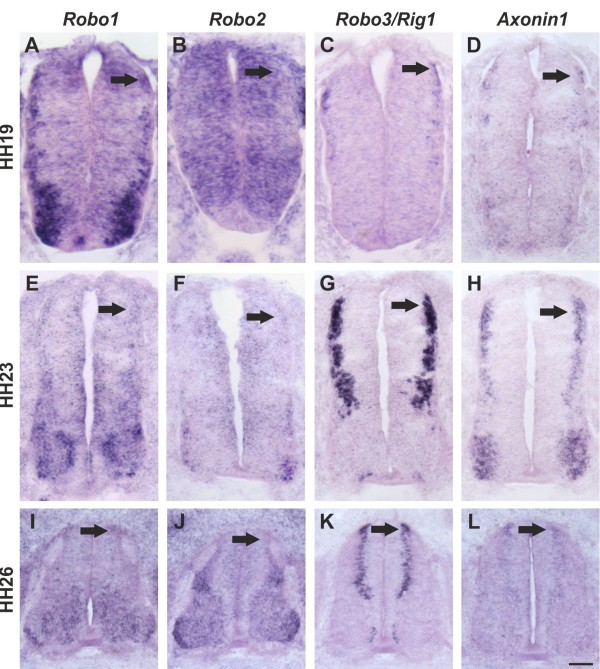
**Commissural axons in the embryonic chicken spinal cord express all three *Robos*.** We used in situ probes specific for *Robo1* (**A**,**E**,**I**), *Robo2* (**B**,**F,J**), and *Robo3* (**C**,**G**,**K**) to assess their expression pattern in comparison to *Axonin-1/TAG-1* (**D**,**H**,**L**) at HH19 (**A**-**D**), HH23 (**E**-**H**), and HH26 (**I**-**L**). *Robo1* mRNA could be detected in commissural neurons as soon as they start to extend axons (arrow in **A**), and continued to be expressed in these neurons throughout the time window of their axonal navigation across the floor plate and into the longitudinal axis (arrows in **E**,**I**). *Robo2* was expressed throughout the neural tube except for the floor plate at HH19 (**B**). Weak expression in a subset of commissural axons was found at HH23 (arrow in **F**). By HH26, *Robo2* mRNA was barely detectable in dorsal commissural neurons (arrow in **J**). At all stages, *Robo3* mRNA was found in dorsolateral commissural neurons (arrows in **C,G,K**). *Axonin-1/TAG-1* expression indicates the position of dorsal commissural neurons (**D**,**H**,**L**). Bars: 33 μm in **A**-**D**, 50 μm in **E**-**H**, 100 μm in **I**-**L**. Dorsal is to the top in all panels.

Based on the similarity of the phenotypes resulting from downregulation of Slit1 and RabGDI, respectively, Robo receptors were likely involved in shifting the balance from positive to negative signals at the floor plate. All three *Robos* are expressed in the chicken spinal cord during the time window of commissural axon pathfinding (Figure 5). *Robo1* is the most widely expressed family member. At HH19, when commissural neurons start to extend axons ventrally, they already express *Robo1* (see also [56]). *Robo1* expression in commissural neurons persisted throughout HH26, the oldest stage analyzed. At that time, commissural neurons have crossed the midline, turned rostrally, and extended some distance along the longitudinal axis. *Robo2* appeared to be expressed only in a subset of commissural neurons when compared to the expression of *Axonin-1/TAG-1*, a marker for dorsolateral commissural neurons (Figure 5). This is consistent with results from mouse [27,28,34] and rat [25], where *Robo1* and *Robo2* expression was found in different subpopulations of dorsal commissural neurons. As in rodents, *Robo3* was expressed almost exclusively in dorsal interneurons including the dorsolateral commissural neurons studied here. The patterns of *Robo3* and *Robo1* expression were largely overlapping, as described in the mouse [28,34]. Thus, *Robo* expression is very similar in commissural neurons in mouse, rat, and chicken. Interestingly, when analyzed at the protein level, precommissural axons expressed high levels of Robo3 but almost no Robo1 on their surface [27,35,56,57]. In contrast, postcommissural axons expressed high levels of Robo1. These findings are consistent with reports from *Drosophila*, where Robo protein was found only on postcommissural axons [16].

### Robos are required for commissural axon navigation across the midline of the embryonic chicken spinal cord

**Figure 6:**
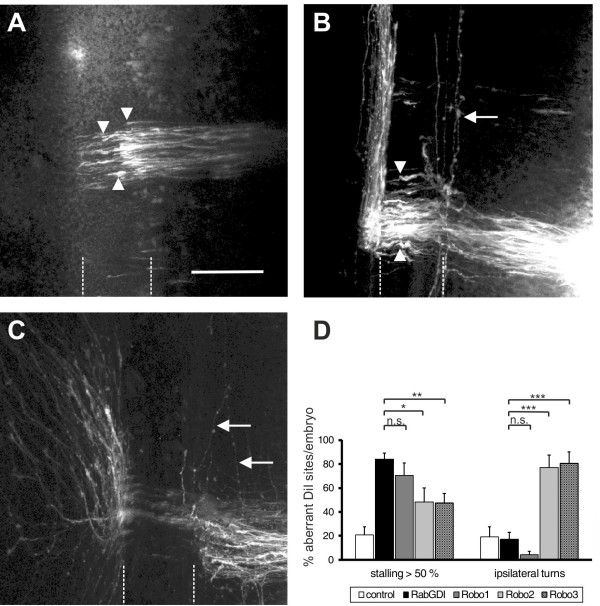
**Loss of *Robo1* function closely mimics the RabGDI phenotype.** In ovo RNAi with dsRNA derived from *Robo1* (**A**), *Robo2* (**B**), and *Robo3* (**C**) was used to silence these genes and compare their effect on commissural axon guidance at the midline. In the absence of Robo1, commissural axons stalled in the floor plate (dashed lines) and failed to reach the contralateral floor-plate border, as seen after loss of *RabGDI* function (arrowheads in **A**, compare to Figure 1C and D). Silencing *Robo2* resulted in a distinct phenotype (**B**). While axons still stalled in the floor plate following loss of Robo2 (arrowheads in **B**), many injection sites additionally showed axons with abnormal ipsilateral turns (arrow in **B**, and **D**). Similarly, loss of *Robo3* function resulted in ipsilateral turns (arrows in **C**). In contrast to the phenotypes seen after downregulation of Robo1 and Robo2, postcommissural axons were strongly defasciculated in the absence of Robo3 and many failed to grow along the contralateral floor-plate border. Phenotype quantifications are shown in (**D**). Significance levels: n.s. not significant, * p<0.05, ** p<0.01, *** p<0.001. Bar: 50 μm.

The induction of loss-of-function phenotypes for the three *Robos* resulted in different effects on the behavior of commissural axons at the floor plate (Figure 6). Loss of *Robo1* function mimicked the *RabGDI* phenotype most closely. Axons grew into the floor plate normally, but failed to reach the contralateral border (Figure 6A; compare to Figure 1C and D). The phenotype was observed at 70.7 ± 10.0% of the injection sites (n = 63, 6 embryos). Silencing *Robo2* or *Robo3* gave less pronounced phenotypes but still resulted in axon stalling at 48.2 ± 11.9% (n = 60, 6 embryos) and 47.5 ± 7.9% of the injection sites (n = 68, 9 embryos), respectively. Detailed analyses of the phenotypes indicated clear differences between the *Robos*. Axons were as likely or even more likely to enter the floor plate in the absence of Robo1 compared to control embryos (Figure 6D), whereas in the absence of Robo2, commissural axons turning ipsilaterally were found at the majority of injection sites (77.1 ± 10.6%; Figure 6B,D). Even more aberrant was the behavior of commissural axons in embryos lacking Robo3 (Figure 6C,D). Many axons turned into the longitudinal axis before they reached the ipsilateral floor-plate border (80.7 ± 9.4% of the injection sites, n = 68, 9 embryos). In addition, the pathfinding behavior of postcommissural axons was strongly affected. Instead of turning rostrally along the floor-plate border, axons either failed to turn or they turned caudally. Even those postcommissural axons that turned rostrally did so in a very unusual manner in the absence of Robo3. Axons displayed a striking defasciculation upon floor-plate exit and did not grow along the floor-plate border. These features were not seen in the absence of either Robo1 or Robo2. Moreover, the deflection phenotype was not observed in the absence of any of the Slits. Taken together, quantitative and qualitative analyses of the loss-of-function phenotypes indicated that loss of *Robo1* function closely resembled the *RabGDI* phenotype.

### The temporal expression of *RabGDI* is consistent with its role in midline crossing of commissural axons

**Figure 7:**
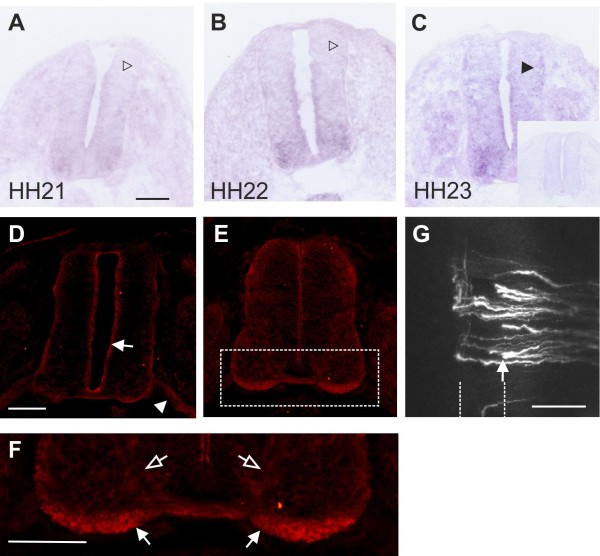
**The temporal expression of *RabGDI* is consistent with its role as a regulator of Robo1 expression on commissural axons.***RabGDI* is not expressed by dorsolateral commissural neurons when their axons grow toward the floor plate between HH19 (not shown) and HH21 (open arrowhead in **A**) or when they have reached the floor plate at HH22 (open arrowhead in **B**). RabGDI is detectable only after commissural axons have entered the floor-plate area at HH23 (arrowhead, **C**). The adjacent section to the one shown in (**C**) was hybridized with the corresponding sense probe as a control (insert in **C**). At HH22, only very low levels of RabGDI protein were detectable in cells lining the central canal (arrow in **D**) and in motor axons (arrowhead in **D**). At HH24, RabGDI protein was localized almost exclusively to post-crossing axons (**E**,**F**; compare levels in post-crossing axons (arrow in **F**) with pre-crossing commissural axons (open arrow in **F**)). Following premature expression of *RabGDI*, many commissural axons failed to enter the floor plate (arrow in **G**), consistent with premature expression of Robo1 on commissural growth cones. Bar 100 μm in **A**-**E** and **G** .

*Robo1* mRNA was expressed in commissural neurons already at HH19, when they start to extend their axons (Figure 5). However, surface expression of Robo1 would be incompatible with midline crossing. And indeed, several studies reported that there was little or no Robo1 protein detected on pre-crossing commissural axons [27,56,57]. Therefore, Robo1 insertion into the membrane of commissural axons appears to be regulated at the post-transcriptional level. RabGDI’s function in vesicle fusion and protein trafficking suggests that it may play a role in regulation of Robo1 surface expression. A prerequisite for this is a tight temporal control of RabGDI expression, which is indeed what we found (Figure 7). *RabGDI* was not expressed during the time when commissural axons extended toward the floor plate (Figure 7A and B) but was clearly detected after commissural axons had entered the floor plate (HH23; Figure 7C). Expression persisted throughout the time window during which commissural axons cross the floorplate turn rostrally and extend along the longitudinal axis of the spinal cord [50] (not shown). In line with these results, we found more RabGDI protein on post-crossing compared to pre-crossing commissural axons at HH24 (Figure 7E,F). Very low levels of RabGDI protein were detected in cells lining the central canal and on motor axons at HH22 (Figure 7D).

Functional evidence supporting the hypothesis that RabGDI controls Robo1 surface expression on commissural axons was found in a series of in vivo experiments, where we expressed RabGDI prematurely (Figure 7G). As expected, expression of *RabGDI* in precommissural axons resulted in stalling *before* floor-plate entry rather than stalling *in* the floor plate, as axons now responded prematurely to the repellent activity of Slit (compare Figure 7G to Figure 1C and D, where axons stall in the floor plate). The effect was seen at 81.1 ± 6.7% of all injection sites (n = 53, 9 embryos).

**Figure 8:**
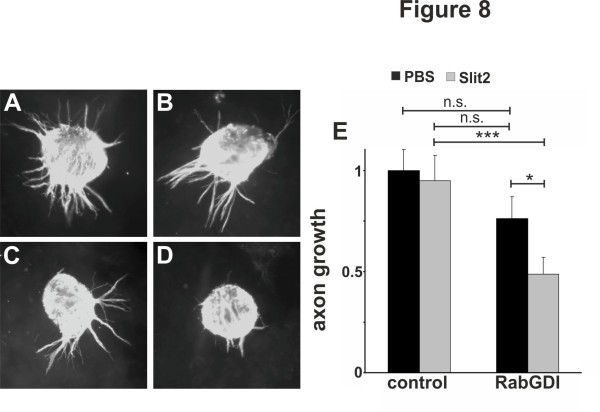
**Pre-crossing commissural axons become sensitive to Slit2 by premature RabGDI expression.** Explants containing young mock-transfected commissural neurons readily extend axons, which correspond to pre-crossing commissural axons, when cultured in a 3D collagen gel (**A**). The addition of recombinant Slit2 does not affect axon growth from these explants (**B**). A slight, but not significant reduction in axon growth was observed when explants were taken from embryos expressing RabGDI prematurely at HH21/22 (see Material and Methods and text for details; **C**,**E**). However, a strong reduction in axon growth was observed when RabGDI-expressing ‘pre-crossing’ commissural axons were cultured in the presence of Slit2 (**D**,**E**). Axons were visualized with anti-neurofilament staining. For quantification of axon growth 20–25 explants per condition were used. Axon length of mock-transfected neurons was set to 1.0 (**E**). Axon length from mock-transfected neurons was not different in the presence of Slit2 (0.95 ± 0.12; p = 0.765). In the absence of Slit2, commissural neurons expressing RabGDI extended axons that were slightly, but not significantly shorter than mock-transfected neurons (0.76 ± 0.11; p = 0.121). In the presence of Slit2, axon length was significantly shorter when neurons expressed RabGDI prematurely (0.49 ± 0.08; p = 0.0028 compared to mock-transfected neurons in the presence of Slit2 and p = 0.048 compared to RabGDI-expressing axons in the absence of Slit).

These in vivo studies are consistent with observations made in vitro. Outgrowth of pre-crossing commissural axons that were precociously expressing RabGDI was inhibited by the presence of recombinant Slit2 in the medium (Figure 8). Again, embryos were electroporated with a plasmid encoding RabGDI at HH18 to express RabGDI prematurely. Explants containing commissural neurons were dissected at HH21/22 and cultured in 3D collagen gels for 1 day in either the absence or presence of Slit2. For a control, commissural neuron explants were prepared from embryos electroporated with the empty vector (Figure 8A,B). As expected, the presence of Slit2 in the medium had no effect on pre-crossing commissural axons (Figure 8B,E). However, premature expression of RabGDI in pre-crossing commissural neurons rendered them sensitive to Slit2 (Figure 8D,E)

These results indicated that RabGDI was not only expressed in the appropriate temporal pattern to influence axon pathfinding in the floor plate, but its expression was indeed also functionally linked to axonal responsiveness to Slit.

### RabGDI is required for insertion of Robo1 into the cell membrane

**Figure 9:**
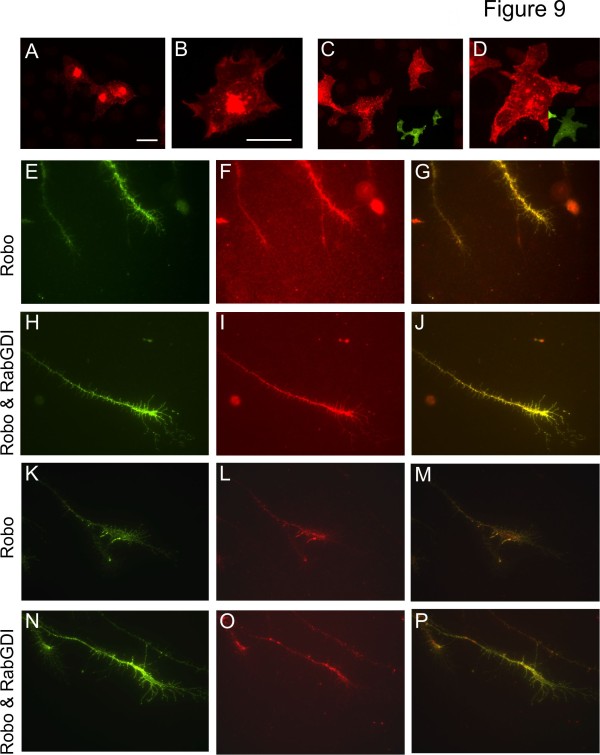
**RabGDI triggers membrane insertion of Robo1.** Robo1 expressed in COS cells was mainly found in the perinuclear area, the endoplasmic reticulum, and the Golgi apparatus (**A** and **B**). Co-transfection of RabGDI resulted in redistribution of Robo1 to vesicular structures in the periphery of the cells (**C** and **D**). Inserts show RabGDI expression. Note that the staining intensity in the perinuclear area was markedly decreased in the presence of RabGDI. To demonstrate surface expression of Robo1 in the presence of RabGDI commissural neurons dissected from HH21/22 chicken embryos expressing either HA-Robo1-myc alone (E-G and K-M) or in combination with RabGDI (H-J and N-P) were grown in culture. Growth cones shown in (**E**-**J**) and (**K**-**P**) were taken from independent experiments. Surface expression of Robo1 was visualized by staining the N-terminal HA-tag before fixation and permeabilization (**E**,**H**,**K**,**N**). The myc staining after fixation and permeabilization revealed total Robo1 levels (**F**,**I**,**L**,**O**). The overlay of both stainings is shown in **G**,**J**,**M** and **P**, respectively. In the presence of RabGDI 27.8% (p = 0.0051) more Robo1 could be detected on the surface of commissural axons. Bar: 10 μm.

The responsiveness of precommissural axons to Slits induced by premature expression of RabGDI suggested a role of RabGDI in Robo1 membrane insertion. Co-expression of Robo1 and RabGDI enhanced insertion of Robo1 into the plasma membrane (Figure 9). In COS cells transfected with myc-tagged Robo1 alone, some Robo1 was found in the plasma membrane but mostly Robo1 localized to the perinuclear area, the endoplasmic reticulum and the Golgi apparatus (Figure 9A,B). When Robo1 was co-transfected with RabGDI, Robo1 was redistributed and staining in the perinuclear area was strongly decreased (Figure 9C,D). More importantly, surface levels of Robo1 on growth cones of commissural axons were dependent on the presence of RabGDI (Figure 9E-P). We expressed Robo1 with an N-terminal HA- and a C-terminal myc-tag in commissural neurons, allowing us to assess the proportion of surface versus total Robo1 protein levels. Embryos were electroporated at HH18 with the tagged Robo1 construct alone or together with RabGDI. At HH21/22, embryos were sacrificed and commissural neurons were cultured for 40 h. Surface Robo1 was detected by staining with the anti-HA antibody prior to fixation. After fixation and permeabilization total Robo1 was stained with anti-myc antibodies. Staining intensities for total Robo1 did not differ between axons originating from embryos electroporated with the tagged Robo1 construct alone and those taken from embryos co-electroporated with RabGDI (p = 0.499). However, when we compared the ratios between surface and total Robo1, we found significantly more Robo1 on the axonal surface in the presence of RabGDI (27.8%; p = 0.0051; Figure 9; see Materials and Methods for details). The finding that total levels of Robo1 protein did not differ is consistent with the presence of high mRNA levels throughout the period of commissural axon development (Figure 5). Furthermore, these results were in agreement with the analysis of Robo1 protein levels in tissue sections, published previously [27,56,57].

**Figure 10:**
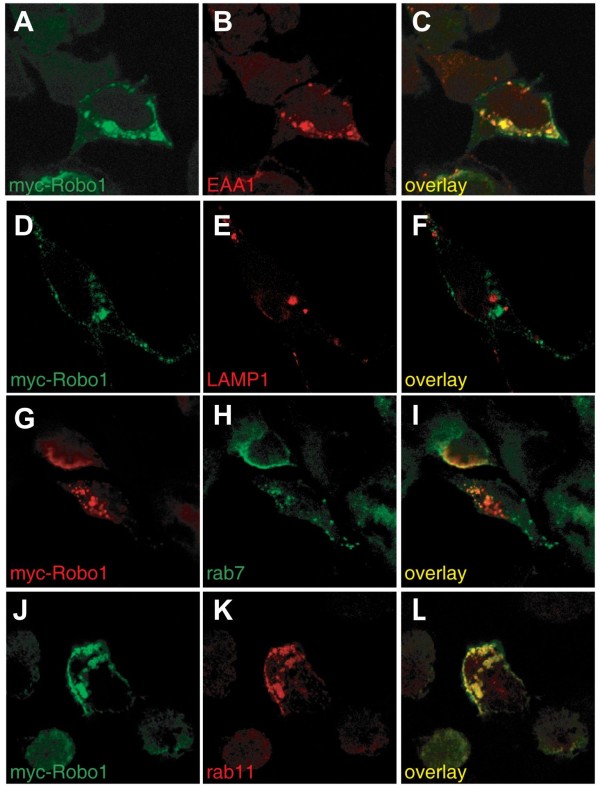
**Robo1 localizes to Rab11-positive vesicles.** Myc-tagged Robo1 was expressed in HEK293 cells. Antibodies recognizing marker proteins for early endosomes (EAA1) and lysosomes (LAMP1), as well as Rab7 (late endosomes) and Rab11 (labeling recycling endosomes) were used to analyze Robo1 localization in the cell. Robo1 (**A**) was found to co-localize considerably with EAA1 (**B** and **C**). No overlap was seen between Robo1 (**D**) and LAMP1 (**EF**). Similarly, Robo1 (**G**) did not co-localize with Rab7 (**HI**), indicating that Robo1 was not shuttled to the late endosome/lysosome compartment. Almost complete overlap was found between Robo1 (**J**) and Rab11 (**KL**), suggesting that Robo1 was predominantly stored in Rab11-positive vesicles which have been shown to be involved in exocytosis and regulated secretion [58,69] .

Our model that RabGDI triggers membrane insertion of Robo1 was further supported by our finding that Robo1 was localized in Rab11-positive vesicles (Figure 10). Rab11 has been shown to label vesicles that are ready to insert their cargo into the plasma membrane [28,58]. In contrast, very little co-localization was seen for Robo1 and Rab7, a marker for the pathway leading to late endosomes and lysosomes.

Taken together, these results indicate that RabGDI is required for the regulation of Robo1 surface expression on commissural axons. On precommissural axons in which RabGDI is absent, Robo1 is not inserted into the membrane but rather stored in vesicles. At the time of growth cone contact with the floor plate, commissural neurons start to express RabGDI, resulting in vesicle fusion and insertion of Robo1 into the growth cone membrane. This, in turn, triggers an increase in Slit responsiveness, causing a shift from positive to negative signals and expulsion of commissural axons from the floor plate.

## Discussion

Midline crossing by commissural axons is regulated by a balance between positive and negative cues [1-3]. In both invertebrates and vertebrates, Slits were identified as repulsive cues associated with the midline [15,27,29] (reviewed in [32]). In order to cross the midline, commissural axons must not express high levels of Robo receptors. However, upregulation of Robo1 is required for commissural axons to leave the floor plate on the contralateral side ([27]; this study). This raises the question how commissural axons are able to achieve the precise temporal regulation of Robo1 expression. In flies, Comm has been shown to keep Robo1 low on axons before they cross the midline [16]. Interestingly, although many aspects of midline crossing have been conserved between vertebrates and invertebrates, no *comm* ortholog has been identified in vertebrates. *Robo1* mRNA is expressed in commissural neurons as soon as they start to extend their axons (Figure 5). However, only very low levels of Robo1 protein have been detected on precommissural axons, suggesting that Robo levels on precommissural axons are regulated at the posttranscriptional or posttranslational level [27,56]. In accordance with these findings, commissural axons were shown to become responsive to Slit only after floor-plate crossing [23]. Recent studies in mice indicate that Robo3 prevents Robo1 from responding to Slit prematurely [28,45]. However, these studies did not explain why almost no Robo1 protein was found on precommissural axons, as opposed to the high levels of Robo1 on postcommissural axons, since mRNA levels remain unchanged in commissural neurons before and after contact with the floor plate.

### RabGDI regulates Robo1 expression on commissural axons

Our in vivo studies support a balance between positive and negative cues derived from growth cone/floor-plate contact that regulates midline crossing (Figure 2). Our results indicate that RabGDI is required for the shift from positive to negative signals. In the absence of RabGDI, commissural axons did not leave the floor plate, presumably because they failed to sense the negative cues required to counteract the positive cues derived from Axonin-1/NrCAM interactions. We have provided evidence supporting this hypothesis by interfering with the positive and negative cues derived from axon/floor plate contact. In support of our model, lowering positive signals partially rescued the failure of commissural axons to leave the floor plate in the absence of RabGDI. As expected, the reverse was also true: lowering negative cues allowed commissural axons to enter the floor plate in the absence of Axonin-1/NrCAM interactions.

Concurrent with previous findings in mice and flies, we demonstrated that the negative cues associated with the midline are Slit1 and Slit2 (Figure 4). As demonstrated biochemically [25] and functionally in vivo and in vitro [27-29,59], Robo receptors bind Slits and mediate their repulsive effects. The absence of Robo1 was found to interfere with commissural axon navigation at the midline. In both, mouse [27] and chicken embryos (this study), commissural axons stalled in the floor plate and failed to leave on the contralateral side. The qualitative and quantitative similarity of the phenotypes observed in the absence of Robo1 and RabGDI suggested that Robo1 might be the receptor that required RabGDI for its expression on the surface of commissural axons. Consistent with the regulation of Robo1 in vivo, commissural neurons expressed RabGDI only when they reached the floor plate (Figure 7; [50]). The upregulation of RabGDI is required for the fusion of Robo1-containing vesicles with the growth cone membrane. The expression of Robo1 on the growth cone surface in turn induces sensitivity to Slits. This shifts the balance toward more negative signals, expelling the growth cone from the floor plate that had initially been perceived as positive. Consistent with this model, commissural axons that expressed RabGDI prematurely, and thus inserted Robo1 into the membrane before floor-plate contact, stalled at the ipsilateral floor-plate border (Figure 7G). These in vivo findings were corroborated by the analysis of Robo1 surface levels in cultured commissural neurons (Figure 9). Premature expression of RabGDI in commissural neurons that had not yet reached the floor plate resulted in a significant increase in Robo1 surface expression without affecting total Robo1 levels. This effect was diminished when commissural neurons of later embryonic stages were cultured, presumably because of the presence of endogenous RabGDI (data not shown). Taken together, these in vivo and in vitro findings clearly support our model that RabGDI expression triggers the insertion of Robo1 into the growth cone membrane at the midline.

So, where does Robo3 fit in? Our loss-of-function studies indicate that Robo3 is required for midline crossing (Figure 6) consistent with observations in mouse [28,45]. Many axons failed to reach the floor plate in the absence of Robo3 (Figure 6C). Those that did were very likely to turn along the ipsilateral floor-plate border instead of crossing the midline. Based on the partial rescue of the midline-crossing defect in mice lacking both *Robo1* and *Robo3*, Sabatier and colleagues concluded that Robo3 might repress the responsiveness of Robo1 to Slit in precommissural axons. Their hypothesis was supported by evidence from explant cultures, where neurons failed to extend axons in the absence of Robo3 in a Slit-dependent manner. More recently, this model was refined by the demonstration that premature responsiveness to Slit was prevented by the Robo3.1 isoform, whereas a different isoform, Robo3.2, was required only after midline crossing. However, neither the originally proposed model suggested by Sabatier and colleagues [28] nor the refined version [45] explains the fact that almost no Robo1 protein is found on pre-crossing commissural axons in contrast to post-crossing commissural axons, although the mRNA is clearly present in commissural neurons at both developmental stages. Furthermore, the *Robo3* loss-of-function phenotype was clearly distinct from the *Robo1* gain-of-function phenotype, as induced by premature RabGDI expression (compare Figures 6C and 7G). Pre-crossing commissural axons expressing Robo1 did not fail to reach the floor plate, but stalled at the ipsilateral floor-plate border (Figure 7G). In contrast, pre-crossing commissural axons lacking Robo3 often failed to reach the floor plate. Furthermore, those axons that managed to cross the floor plate showed very aberrant behavior upon floor-plate exit, consistent with the suggestion that Robo3 (Robo3.2) may have a role in post-crossing commissural axon guidance [45].

Our findings are in agreement with the suggested role of Robo3 in repressing Robo1’s response to Slit, but they suggest that there is an additional mechanism involved in the regulation of Robo1’s effect on midline crossing by commissural axons. Based on the conclusions drawn from the analysis of mice lacking *Robo1* and *Robo3* as well as our own analysis we suggest the following model for commissural axon guidance: Pre-crossing commissural axons are attracted toward the floor plate by the long-range attractant Netrin-1. They express Axonin-1 and Robo3, but no RabGDI, and therefore very low levels of Robo1 on their surface. Before midline contact, Robo1’s responsiveness to Slit is blocked by Robo3.1. The interaction between Axonin-1 and NrCAM expressed by the floor plate makes precommissural axons enter the floor plate. The contact between the growth cone and the floor plate triggers the expression of RabGDI. This in turn is required for vesicle fusion at the growth cone. Robo1 that was stored in these vesicles is inserted into the membrane. Increasing amounts of Robo1 enhance the responsiveness of the commissural growth cones to negative cues and, thus, expel the growth cone from the floor plate.

### Multiple mechanisms contribute to the regulation of commissural axons’ switch in responsiveness

In mouse, the switch from attraction to repulsion was shown to include Semaphorin3B [23,60,61]. Pre-crossing commissural axons were kept unresponsive to Sema3B by proteolytic cleavage of the receptor component PlexinA1, which, together with Neuropilin-2, mediates repulsion in response to Sema3B [60]. NrCAM derived from the floor plate by an unknown release mechanism was found to suppress PlexinA1 cleavage and therefore induce a switch in commissural axons’ behavior. In addition, in cultured rat commissural neurons Shh was shown to contribute to the gain in responsiveness to Semaphorins [61]. Shh-induced changes in cAMP levels were suggested as the underlying mechanism.

Due to the accessibility of the chicken embryo for in vivo manipulations during neural development a separation of the effects on pre- versus post-crossing commissural axon guidance along the longitudinal axis of the spinal cord is possible. In chick, Shh was shown to affect post-crossing commissural axon guidance along the contralateral floor-plate border both directly and indirectly [62]. Post-crossing commissural axons were rendered sensitive to the repellent activity of Shh by Hhip [51]. In addition, Shh indirectly regulated the attractive activity of Wnt5a and Wnt7a by inducing a gradient of the soluble Wnt antagonist Sfrp (secreted Frizzled-related protein) [62].

Thus, taken together, different molecular mechanisms contribute to the switch in axonal responsiveness at the floor plate. Changes occur at the transcriptional level, as shown for Hhip in Shh signaling [51], and at the post-translational level. An important contributor to the change in axonal behavior is the gain in Robo1-dependent responsiveness to Slits. Thus, surface levels of Robo1 have to be tightly regulated. RabGDI is a highly conserved regulator of vesicle trafficking. The identity between chicken and human RabGDI is 87% at the amino acid level. In fact, flies also express a RabGDI ortholog with 68% identity to chicken and human RabGDI. Therefore, the contribution of RabGDI to the regulation of axon guidance receptors on the growth cone surface at choice points is very likely a general mechanism in vertebrates. It remains to be shown whether RabGDI-dependent vesicle trafficking also plays a role in axonal behavior at choice points in invertebrates.

## Conclusion

Upregulation of Robo surface expression is required for commissural axons to leave the midline area. In *Drosophila* Comm was shown to be crucial in this process, but to date, no vertebrate ortholog has been found. Here, we show that in vertebrates RabGDI controls midline crossing by regulating surface levels of Robo1 post-translationally. Thus, the regulatory mechanism is conserved between flies and vertebrates but depends on different molecules: flies use Comm, whereas vertebrates use RabGDI to regulate surface expression of Robo1.

## Methods

### In ovo RNAi

All experiments with chicken embryos were carried out in accordance with the guidelines of the Cantonal Veterinary Office of Zurich. Fertilized eggs obtained from a local supplier were windowed after 3 days of incubation at 38.5°C. To get access to the embryo for experimental manipulations, extra-embryonic membranes were carefully removed. Following injection into the central canal of long double-stranded RNA (dsRNA; 100–300 ng/μl) together with a plasmid encoding YFP or GFP under the control of the chicken β-actin promoter at HH18/19 [63], transfection was achieved by electroporation, as described previously [50]. After 2 days of incubation, embryos were sacrificed at HH25/26 and the trajectory of commissural axons was analyzed in an open-book configuration by application of DiI (Molecular Probes), as described previously [64]. Efficiency and accuracy of electroporation were assessed by YFP or GFP expression.

The specific downregulation of the target genes was verified in all cases by using two independent, non-overlapping fragments of cDNA for the generation of dsRNA. The sequences used were: bp 763–1157 (ORF) and bp 1787–2206 (3’-UTR) for *RabGDI* (AF076291), bp 568–770 and bp 878-1789/3061-3348 for *Robo1* (XM_416673), bp 79–626 and bp 626–1565 for *Robo2* (AF364048), bp 569–807 and bp 808–1018 for *Robo3* (XM_425794), bp 2142–2541 and bp 2541–3995 for *Slit1* (XM_421715), bp 3155–3946 and bp 5892–6394 for *Slit2* (XM_001232040) and bp 3159–3832 and bp 3832–4572 for *Slit3* (XM_414503). Details on dsRNA derived from Axonin-1 and NrCAM are given in [50].

### Quantification of stalling and ipsilateral turns

A minimum of six embryos was analyzed for each condition. The quantification of phenotypes was done by a person blind to the experimental condition. Only DiI injections sites that were in the appropriate location in the dorsal-most part of the spinal cord were included in the analysis. As it was impossible to count axons at individual injection sites, the percentage of axons stalling in or at the ipsilateral border of the floor plate was estimated, and the injection site was classified as showing no, a weak, or a strong phenotype depending on whether 0–10, 10–50, or more than 50% of the axons stalled, respectively. For each open book, only the number of injection sites with at least 50% of the labeled axons not reaching the contralateral floor plate border (representing a strong phenotype) was considered for the quantitative analysis. As a separate parameter, the number of injection sites with axons turning ipsilaterally was determined. Mean and SEM (standard error of the mean) were calculated and subjected to statistical analyses (Student’s *t*-test, ANOVA for comparison of more than two groups, followed by Bonferroni correction on vassarstats.net).

### Quantitative real time PCR

Spinal cords were removed from control and experimental embryos after in ovo RNAi between HH28 and HH30 [63], transferred to RNAlater (Ambion), and immediately frozen in liquid nitrogen. Total RNA was extracted using the RNAeasy Mini extraction kit (Promega) according to the manufacturer’s protocol. Quantitative RT-PCR was carried out with the SuperScript III Platinum Two-step qRT-PCR kit (Invitrogen) on AB 7900 HT from Applied Biosystems using the SYBR green PCR master mix (Applied Biosystems) to monitor double-stranded DNA. Primers for *Robo1* were designed with Primer3 (freeware); 5’- AGT GAC TTT CCA GTG TGA AGC AAC −3’ (forward), 5’- GTG ATG GTG AGG TCT CCT GTC TG −3’ (reverse). The 200-bp long PCR product was normalized to levels of *GAPDH*, 18s RNA, and *β-actin*. RNA was prepared from three independent experiments, and measurements were performed in triplicates. Using this approach, a downregulation of *Robo1* mRNA by more than 30% was achieved in all experiments

### Transient transfection of HEK293 and COS cells

HEK293 or COS cells were grown on poly-L-lysine (Sigma) coated coverslips for 42 h after transfection with plasmids and dsRNA using the calcium phosphate method. Cells were fixed with 4% paraformaldehyde and myc-tagged Robo1 was detected using either the monoclonal antibody 9E10 (Developmental Studies Hybridoma Bank) or a rabbit antibody raised against the myc tag (Abcam). Marker proteins for early (EAA1) and late endosomes (LAMP1; Rab7) were used to demonstrate localization of Robo1. Antibodies were obtained from Abcam (EAA1, Rab7, Rab11) or were a gift from U. Greber (LAMP1). For downregulation of RabGDI the long dsRNA derived from the RabGDI cDNA was cut to short fragments in vitro by RNAse III (New England Biolabs) to avoid unspecific effects on protein synthesis known to occur in cell lines after transfection with long dsRNA.

### In situ hybridization

In situ hybridization was carried out essentially as described previously [52] using digoxigenin-labeled in situ probes (Roche Diagnostics). The probes for *Robo1*, *Robo2*, *Slit1*, *Slit2*, and *Slit3* were generated as described before using plasmids kindly provided by Dr. Ed Laufer [65]. The probe for detection of chicken *Robo3* was prepared using a cDNA fragment obtained from Dr. Avihu Klar.

### Immunohistochemistry and Western blotting

Staining of 20-μm-thick cryostat sections was carried out as described previously [66]. Western blots were performed as specified elsewhere [67]. Antibodies used in this study were: rabbit anti-GFP (recognizing also YFP; Abcam), rabbit anti-HA (Rockland), mouse anti-myc (9E10; Developmental Studies Hybridoma Bank), rabbit anti-GDI (Zymed), and HRP-coupled sheep anti-rabbit IgG (Cappel). Fluorophore-conjugated secondary antibodies were purchased from Molecular Probes.

### Ectopic expression of RabGDI and Robo1

For gain-of-function experiments the open-reading frame of *RabGDI* was cloned into pcDNA3.1 (Invitrogen) and into a plasmid derived from pIRES (Clontech), where the CMV promoter was exchanged for the chicken β-actin promoter [51]. Injections of both plasmids resulted in the same phenotype. A myc-tagged Robo1 (obtained from V. Sundaresan) was subcloned with an N-terminal HA- tag into pCAGGS (kindly provided by S. Arber).

### Surface localization of Robo1 in commissural explants

Dorsal spinal cords of HH18 chicken embryos were electroporated with HA-Robo1-myc alone, or together with the pcDNA3.1-RabGDI expression construct. Commissural explants of electroporated HH21/22 embryos were cultured for 40h as previously described [21]. For staining of surface Robo1 explants were incubated with the anti-HA antibody prior to fixation for 1h at 37°C. After fixation and permeabilization the anti-myc antibody was added. The secondary antibodies were added simultaneously. Staining intensities were measured using ImageJ. A total of 60 neurites from 10 embryos were analyzed per condition. For quantification of surface Robo1 the staining intensity of the HA-tag was measured and expressed as percentage of the myc-staining intensity (total Robo1). Values obtained for neurons expressing the HA-Robo1-myc construct alone were compared to neurons co-expressing RabGDI. The average pixel values for total Robo1 (myc staining) did not differ between the two conditions [67.84 (Robo1 alone) and 65.15 (Robo and GDI); p = 0.583].

### Slit responsiveness of pre-crossing commissural axons

To test for the requirement of RabGDI in rendering commissural axons sensitive to Slit, we cultured explants of dorsal spinal cords in 3D collagen gels as described previously [62]. Embryos were electroporated at HH18 with an empty vector (for control explants) or with a vector encoding RabGDI. Explants were prepared from HH21/22 embryos and cultured in the absence or presence of recombinant Slit2 (R&D Systems, cat. no. 5444-SL). Axons were visualized with anti-neurofilament (RMO270) and anti-axonin-1 antibodies. Axon growth was quantified as described previously [62]. For each condition, 20–25 explants from 3 independent experiments were analyzed.

## Abbreviations

Comm: Commissureless; dsRNA: Double-stranded RNA; E: Embryonic day; GEF: Guanine nucleotide exchange factor; HH: Hamburger and Hamilton stage; ORF: Open reading frame; RabGDI: Rab Guanine Nucleotide Dissociation Inhibitor; RNAi: RNA interference; Shh: Sonic hedgehog; YFP: Yellow fluorescent protein.

## Competing interests

The author(s) declare that they have no competing interests.

## Authors’ contributions

MP, VN, MD, TA, BK, and ES carried out the experiments, analyzed the data, and prepared the figures. VN and ES wrote the manuscript. ES conceived the study. All authors read and approved the final manuscript.

## Supplementary Material

Additional file 1 **Figure S1.** Dorsal electroporation of dsRabGDI results in the same phenotype as one-sided electroporation. Targeting dsRNA derived from RabGDI only into the dorsal spinal cord reproduced the phenotype seen after electroporation of one side of the spinal cord (A). A majority of growth cones stalled in the floor plate (arrows in B). Aberrant axonal pathfinding was observed at 72.3% of the injection sites after dorsal targeting, compared to 70.6% after unilateral targeting. Dorsal targeting of an EGFP-expression plasmid did not interfere with axon guidance. All axons had crossed the floor plate by HH25 (C and D). Bar 100 μm. Click here for file

Additional file 2 **Figure S2.** Downregulation of RabGDI does not interfere with spinal cord patterning. Transverse sections of HH23 embryos were stained with Pax3 (A and B), Nkx2.2 (C and D), and Isl-1 (E and F) to assess spinal cord patterning. The electroporation of dsRabGDI did not change the expression of Pax3 (B), Nkx2.2 (D), or Isl-1 (F). Inserts in B,D, and F show EGFP expression from a co-injected plasmid. A, C, and E show sections taken from a non-injected, age-matched control embryo. Click here for file

## References

[B1] Tessier-Lavigne M Goodman CS The molecular biology of axon guidance Science 1996 274 1123 1133 10.1126/science.274.5290.1123 8895455

[B2] Dickson BJ Molecular mechanisms of axon guidance Science 2002 298 1959 1964 10.1126/science.1072165 12471249

[B3] Stoeckli ET Landmesser LT Axon guidance at choice points Curr Opin Neurobiol 1998 8 73 79 10.1016/S0959-4388(98)80010-X 9568394

[B4] Kaprielian Z Runko E Imondi R Axon guidance at the midline choice point Dev Dyn 2001 221 154 181 10.1002/dvdy.1143 11376484

[B5] Chisholm A Tessier-Lavigne M Conservation and divergence of axon guidance mechanisms Curr Opin Neurobiol 1999 9 603 615 10.1016/S0959-4388(99)00021-5 10508749

[B6] Araujo SJ Tear G Axon guidance mechanisms and molecules: lessons from invertebrates Nat Rev Neurosci 2003 4 910 922 14595402 10.1038/nrn1243

[B7] Augsburger A Schuchardt A Hoskins S Dodd J Butler S BMPs as mediators of roof plate repulsion of commissural neurons Neuron 1999 24 127 141 10.1016/S0896-6273(00)80827-2 10677032

[B8] Butler SJ Dodd J A role for BMP heterodimers in roof plate-mediated repulsion of commissural axons Neuron 2003 38 389 401 10.1016/S0896-6273(03)00254-X 12741987

[B9] Islam SM Shinmyo Y Okafuji T Su Y Naser IB Ahmed G Zhang S Chen S Ohta K Kiyonari H Draxin, a repulsive guidance protein for spinal cord and forebrain commissures Science 2009 323 388 393 10.1126/science.1165187 19150847

[B10] Charron F Tessier-Lavigne M Novel brain wiring functions for classical morphogens: a role as graded positional cues in axon guidance Development 2005 132 2251 2262 10.1242/dev.01830 15857918

[B11] Tear G Axon guidance at the central nervous system midline Cell Mol Life Sci 1999 55 1365 1376 10.1007/s000180050377 10518986 PMC11146891

[B12] Seeger M Tear G Ferres-Marco D Goodman CS Mutations affecting growth cone guidance in Drosophila: genes necessary for guidance toward or away from the midline Neuron 1993 10 409 426 10.1016/0896-6273(93)90330-T 8461134

[B13] Tear G Harris R Sutaria S Kilomanski K Goodman CS Seeger MA commissureless controls growth cone guidance across the CNS midline in Drosophila and encodes a novel membrane protein Neuron 1996 16 501 514 10.1016/S0896-6273(00)80070-7 8785048

[B14] Kidd T Brose K Mitchell KJ Fetter RD Tessier-Lavigne M Goodman CS Tear G Roundabout controls axon crossing of the CNS midline and defines a novel subfamily of evolutionarily conserved guidance receptors Cell 1998 92 205 215 10.1016/S0092-8674(00)80915-0 9458045

[B15] Kidd T Bland KS Goodman CS Slit is the midline repellent for the robo receptor in Drosophila Cell 1999 96 785 794 10.1016/S0092-8674(00)80589-9 10102267

[B16] Kidd T Russell C Goodman CS Tear G Dosage-sensitive and complementary functions of roundabout and commissureless control axon crossing of the CNS midline Neuron 1998 20 25 33 10.1016/S0896-6273(00)80431-6 9459439

[B17] Myat A Henry P McCabe V Flintoft L Rotin D Tear G Drosophila Nedd4, a ubiquitin ligase, is recruited by Commissureless to control cell surface levels of the roundabout receptor Neuron 2002 35 447 459 10.1016/S0896-6273(02)00795-X 12165468

[B18] Keleman K Rajagopalan S Cleppien D Teis D Paiha K Huber LA Technau GM Dickson BJ Comm sorts robo to control axon guidance at the Drosophila midline Cell 2002 110 415 427 10.1016/S0092-8674(02)00901-7 12202032

[B19] Keleman K Ribeiro C Dickson BJ Comm function in commissural axon guidance: cell-autonomous sorting of Robo in vivo Nat Neurosci 2005 8 156 163 10.1038/nn1388 15657595

[B20] Stoeckli ET Landmesser LT Axonin-1, Nr-CAM, and Ng-CAM play different roles in the in vivo guidance of chick commissural neurons Neuron 1995 14 1165 1179 10.1016/0896-6273(95)90264-3 7541632

[B21] Stoeckli ET Sonderegger P Pollerberg GE Landmesser LT Interference with axonin-1 and NrCAM interactions unmasks a floor-plate activity inhibitory for commissural axons Neuron 1997 18 209 221 10.1016/S0896-6273(00)80262-7 9052792

[B22] Fitzli D Stoeckli ET Kunz S Siribour K Rader C Kunz B Kozlov SV Buchstaller A Lane RP Suter DM Dreyer WJ Sonderegger P A direct interaction of axonin-1 with NgCAM-related cell adhesion molecule (NrCAM) results in guidance, but not growth of commissural axons J Cell Biol 2000 149 951 968 10.1083/jcb.149.4.951 10811834 PMC2174557

[B23] Zou Y Stoeckli E Chen H Tessier-Lavigne M Squeezing axons out of the gray matter: a role for slit and semaphorin proteins from midline and ventral spinal cord Cell 2000 102 363 375 10.1016/S0092-8674(00)00041-6 10975526

[B24] Battye R Stevens A Jacobs JR Axon repulsion from the midline of the Drosophila CNS requires slit function Development 1999 126 2475 2481 10226006 10.1242/dev.126.11.2475

[B25] Brose K Bland KS Wang KH Arnott D Henzel W Goodman CS Tessier-Lavigne M Kidd T Slit proteins bind Robo receptors and have an evolutionarily conserved role in repulsive axon guidance Cell 1999 96 795 806 10.1016/S0092-8674(00)80590-5 10102268

[B26] Li HS Chen JH Wu W Fagaly T Zhou L Yuan W Dupuis S Jiang ZH Nash W Gick C Ornitz DM Wu JY Rao Y Vertebrate slit, a secreted ligand for the transmembrane protein roundabout, is a repellent for olfactory bulb axons Cell 1999 96 807 818 10.1016/S0092-8674(00)80591-7 10102269

[B27] Long H Sabatier C Ma L Plump A Yuan W Ornitz DM Tamada A Murakami F Goodman CS Tessier-Lavigne M Conserved roles for Slit and Robo proteins in midline commissural axon guidance Neuron 2004 42 213 223 10.1016/S0896-6273(04)00179-5 15091338

[B28] Sabatier C Plump AS Le M Brose K Tamada A Murakami F Lee EY Tessier-Lavigne M The divergent Robo family protein rig-1/Robo3 is a negative regulator of slit responsiveness required for midline crossing by commissural axons Cell 2004 117 157 169 10.1016/S0092-8674(04)00303-4 15084255

[B29] Hammond R Vivancos V Naeem A Chilton J Mambetisaeva E Andrews W Sundaresan V Guthrie S Slit-mediated repulsion is a key regulator of motor axon pathfinding in the hindbrain Development 2005 132 4483 4495 10.1242/dev.02038 16162649

[B30] Holmes GP Negus K Burridge L Raman S Algar E Yamada T Little MH Distinct but overlapping expression patterns of two vertebrate Slit homologs implies functional roles in CNS development and organogenesis Mech Dev 1998 79 57 72 10.1016/S0925-4773(98)00174-9 10349621

[B31] Itoh A Miyabayashi T Ohno M Sakano S Cloning and expressions of three mammalian homologues of Drosophila slit suggest possible roles for Slit in the formation and maintenance of the nervous system Brain Res Mol Brain Res 1998 62 175 186 9813312 10.1016/s0169-328x(98)00224-1

[B32] Wong K Park HT Wu JY Rao Y Slit proteins: molecular guidance cues for cells ranging from neurons to leukocytes Curr Opin Genet Dev 2002 12 583 591 10.1016/S0959-437X(02)00343-X 12200164

[B33] Yuan W Zhou L Chen JH Wu JY Rao Y Ornitz DM The mouse SLIT family: secreted ligands for ROBO expressed in patterns that suggest a role in morphogenesis and axon guidance Dev Biol 1999 212 290 306 10.1006/dbio.1999.9371 10433822

[B34] Camurri L Mambetisaeva E Sundaresan V Rig-1 a new member of Robo family genes exhibits distinct pattern of expression during mouse development Gene Expr Patterns 2004 4 99 103 10.1016/S1567-133X(03)00142-X 14678835

[B35] Sundaresan V Mambetisaeva E Andrews W Annan A Knoll B Tear G Bannister L Dynamic expression patterns of Robo (Robo1 and Robo2) in the developing murine central nervous system J Comp Neurol 2004 468 467 481 10.1002/cne.10984 14689480

[B36] Huminiecki L Gorn M Suchting S Poulsom R Bicknell R Magic roundabout is a new member of the roundabout receptor family that is endothelial specific and expressed at sites of active angiogenesis Genomics 2002 79 547 552 10.1006/geno.2002.6745 11944987

[B37] Weitzman M Bayley EB Naik UP Robo4: a guidance receptor that regulates angiogenesis Cell Adh Migr 2008 2 220 222 10.4161/cam.2.4.7061 19270536 PMC2637482

[B38] Park KW Morrison CM Sorensen LK Jones CA Rao Y Chien CB Wu JY Urness LD Li DY Robo4 is a vascular-specific receptor that inhibits endothelial migration Dev Biol 2003 261 251 267 10.1016/S0012-1606(03)00258-6 12941633

[B39] Bedell VM Yeo SY Park KW Chung J Seth P Shivalingappa V Zhao J Obara T Sukhatme VP Drummond IA roundabout4 is essential for angiogenesis in vivo Proc Natl Acad Sci USA 2005 102 6373 6378 10.1073/pnas.0408318102 15849270 PMC1088354

[B40] Chien CB Why does the growth cone cross the road? Neuron 1998 20 3 6 10.1016/S0896-6273(00)80427-4 9459435

[B41] Guthrie S Axon guidance: mice and men need Rig and Robo Curr Biol 2004 14 R632 R634 10.1016/j.cub.2004.07.050 15296783

[B42] Garbe DS Bashaw GJ Axon guidance at the midline: from mutants to mechanisms Crit Rev Biochem Mol Biol 2004 39 319 341 10.1080/10409230490906797 15763708

[B43] Dickson BJ Gilestro GF Regulation of commissural axon pathfinding by slit and its Robo receptors Annu Rev Cell Dev Biol 2006 22 651 675 10.1146/annurev.cellbio.21.090704.151234 17029581

[B44] Georgiou M Tear G Commissureless is required both in commissural neurones and midline cells for axon guidance across the midline Development 2002 129 2947 2956 12050141 10.1242/dev.129.12.2947

[B45] Chen Z Gore BB Long H Ma L Tessier-Lavigne M Alternative splicing of the Robo3 axon guidance receptor governs the midline switch from attraction to repulsion Neuron 2008 58 325 332 10.1016/j.neuron.2008.02.016 18466743

[B46] Seabra MC Mules EH Hume AN Rab GTPases, intracellular traffic and disease Trends Mol Med 2002 8 23 30 10.1016/S1471-4914(01)02227-4 11796263

[B47] Pfeffer S Aivazian D Targeting Rab GTPases to distinct membrane compartments Nat Rev Mol Cell Biol 2004 5 886 896 15520808 10.1038/nrm1500

[B48] D’Adamo P Menegon A Lo Nigro C Grasso M Gulisano M Tamanini F Bienvenu T Gedeon AK Oostra B Wu SK Tandon A Valtorta F Balch WE Chelly J Toniolo D Mutations in GDI1 are responsible for X-linked non-specific mental retardation Nat Genet 1998 19 134 139 10.1038/487 9620768

[B49] D’Adamo P Welzl H Papadimitriou S Raffaele Di Barletta M Tiveron C Tatangelo L Pozzi L Chapman PF Knevett SG Ramsay MF Valtorta F Leoni C Menegon A Wolfer DP Lipp HP Toniolo D Deletion of the mental retardation gene Gdi1 impairs associative memory and alters social behavior in mice Hum Mol Genet 2002 11 2567 2580 10.1093/hmg/11.21.2567 12354782

[B50] Pekarik V Bourikas D Miglino N Joset P Preiswerk S Stoeckli ET Screening for gene function in chicken embryo using RNAi and electroporation Nat Biotechnol 2003 21 93 96 10.1038/nbt770 12496763

[B51] Bourikas D Pekarik V Baeriswyl T Grunditz A Sadhu R Nardo M Stoeckli ET Sonic hedgehog guides commissural axons along the longitudinal axis of the spinal cord Nat Neurosci 2005 8 297 304 10.1038/nn1396 15746914

[B52] Mauti O Sadhu R Gemayel J Gesemann M Stoeckli ET Expression patterns of plexins and neuropilins are consistent with cooperative and separate functions during neural development BMC Dev Biol 2006 6 32 10.1186/1471-213X-6-32 16846494 PMC1543641

[B53] Simpson JH Bland KS Fetter RD Goodman CS Short-range and long-range guidance by Slit and its Robo receptors: a combinatorial code of Robo receptors controls lateral position Cell 2000 103 1019 1032 10.1016/S0092-8674(00)00206-3 11163179

[B54] Bagri A Marin O Plump AS Mak J Pleasure SJ Rubenstein JL Tessier-Lavigne M Slit proteins prevent midline crossing and determine the dorsoventral position of major axonal pathways in the mammalian forebrain Neuron 2002 33 233 248 10.1016/S0896-6273(02)00561-5 11804571

[B55] Plump AS Erskine L Sabatier C Brose K Epstein CJ Goodman CS Mason CA Tessier-Lavigne M Slit1 and Slit2 cooperate to prevent premature midline crossing of retinal axons in the mouse visual system Neuron 2002 33 219 232 10.1016/S0896-6273(01)00586-4 11804570

[B56] Mambetisaeva ET Andrews W Camurri L Annan A Sundaresan V Robo family of proteins exhibit differential expression in mouse spinal cord and Robo-Slit interaction is required for midline crossing in vertebrate spinal cord Dev Dyn 2005 533 41 51 15768400 10.1002/dvdy.20324

[B57] Reeber SL Sakai N Nakada Y Dumas J Dobrenis K Johnson JE Kaprielian Z Manipulating Robo expression in vivo perturbs commissural axon pathfinding in the chick spinal cord J Neurosci 2008 28 8698 8708 10.1523/JNEUROSCI.1479-08.2008 18753371 PMC2886497

[B58] Ward ES Martinez C Vaccaro C Zhou J Tang Q Ober RJ From sorting endosomes to exocytosis: association of Rab4 and Rab11 GTPases with the Fc receptor, FcRn, during recycling Mol Biol Cell 2005 16 2028 2038 10.1091/mbc.E04-08-0735 15689494 PMC1073680

[B59] Marillat V Sabatier C Failli V Matsunaga E Sotelo C Tessier-Lavigne M Chedotal A The slit receptor Rig-1/Robo3 controls midline crossing by hindbrain precerebellar neurons and axons Neuron 2004 43 69 79 10.1016/j.neuron.2004.06.018 15233918

[B60] Nawabi H Briancon-Marjollet A Clark C Sanyas I Takamatsu H Okuno T Kumanogoh A Bozon M Takeshima K Yoshida Y A midline switch of receptor processing regulates commissural axon guidance in vertebrates Genes Dev 2010 24 396 410 10.1101/gad.542510 20159958 PMC2816738

[B61] Parra LM Zou Y Sonic hedgehog induces response of commissural axons to Semaphorin repulsion during midline crossing Nat Neurosci 2010 13 29 35 10.1038/nn.2457 19946319

[B62] Domanitskaya E Wacker A Mauti O Baeriswyl T Esteve P Bovolenta P Stoeckli ET Sonic hedgehog guides post-crossing commissural axons both directly and indirectly by regulating Wnt activity J Neurosci 2010 30 11167 11176 10.1523/JNEUROSCI.1488-10.2010 20720124 PMC6633477

[B63] Hamburger V Hamilton HL A series of normal stages in the development of the chick embryo J Morphol 1951 88 49 92 10.1002/jmor.1050880104 1304821

[B64] Perrin FE Stoeckli ET Use of lipophilic dyes in studies of axonal pathfinding in vivo Microsc Res Tech 2000 48 25 31 10.1002/(SICI)1097-0029(20000101)48:1<25::AID-JEMT4>3.0.CO;2-F 10620782

[B65] Vargesson N Luria V Messina I Erskine L Laufer E Expression patterns of Slit and Robo family members during vertebrate limb development Mech Dev 2001 106 175 180 10.1016/S0925-4773(01)00430-0 11472852

[B66] Perrin FE Rathjen FG Stoeckli ET Distinct subpopulations of sensory afferents require F11 or axonin-1 for growth to their target layers within the spinal cord of the chick Neuron 2001 30 707 723 10.1016/S0896-6273(01)00315-4 11430805

[B67] Niederkofler V Salie R Sigrist M Arber S Repulsive guidance molecule (RGM) gene function is required for neural tube closure but not retinal topography in the mouse visual system J Neurosci 2004 24 808 818 10.1523/JNEUROSCI.4610-03.2004 14749425 PMC6729817

[B68] De Bellard ME Rao Y Bronner-Fraser M Dual function of Slit2 in repulsion and enhanced migration of trunk, but not vagal, neural crest cells J Cell Biol 2003 162 269 279 10.1083/jcb.200301041 12876276 PMC2172792

[B69] Ng EL Tang BL Rab GTPases and their roles in brain neurons and glia Brain Res Rev 2008 58 236 246 10.1016/j.brainresrev.2008.04.006 18485483

