# Supplementary material for: RabGDI controls axonal midline crossing by regulating Robo1 surface expression
Source: Neural Dev. 2012 Nov 9;7:36. doi: 10.1186/1749-8104-7-36 (PMC3520763; doi:10.1186/1749-8104-7-36)
Supplement: Additional file 1 — Figure S1. Dorsal electroporation of dsRabGDI results in the same phenotype as one-sided electroporation. Targeting dsRNA derived from RabGDI only into the dorsal spinal cord reproduced the phenotype seen after electroporation of one side of the spinal cord (A). A majority of growth cones stalled in the floor plate (arrows in B). Aberrant axonal pathfinding was observed at 72.3% of the injection sites after dorsal targeting, compared to 70.6% after unilateral targeting. Dorsal targeting of an EGFP-expression plasmid did not interfere with axon guidance. All axons had crossed the floor plate by HH25 (C and D). Bar 100 μm. [file 1749-8104-7-36-S1.pdf]

Additional Figure 1

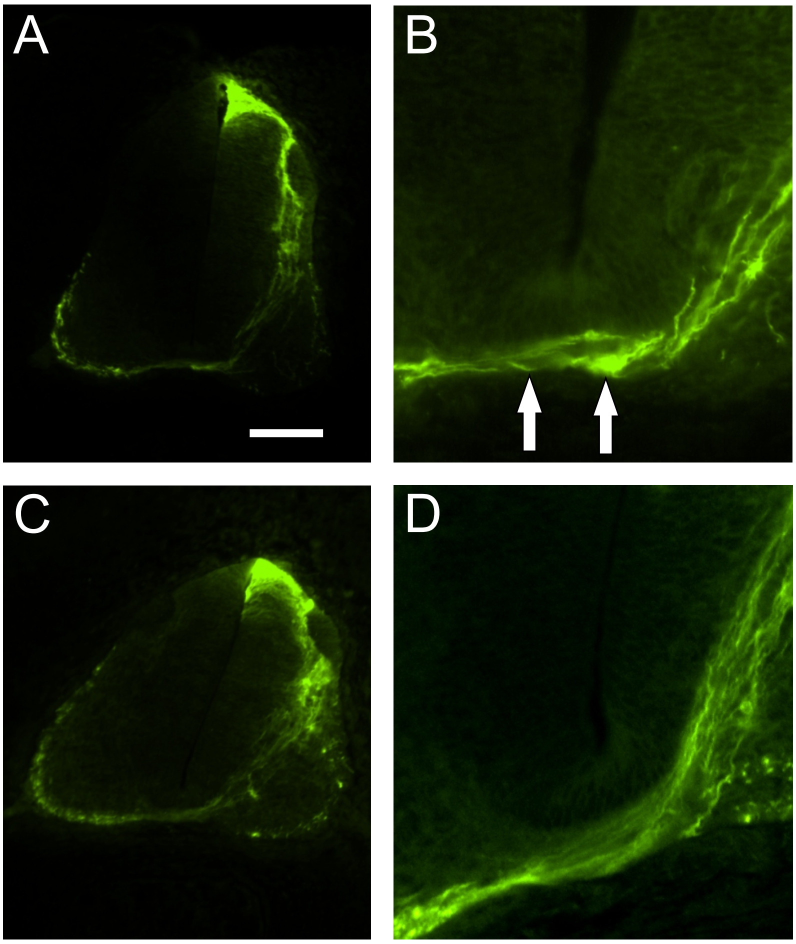

Dorsal electroporation of dsRabGDI results in the same phenotype as one-sided electroporation.

Targeting dsRNA derived from RabGDI only into the dorsal spinal cord reproduced the phenotype seen after electroporation of one side of the spinal cord (**A).** A majority of growth cones stalled in the floor plate (arrows in **B**). Aberrant axonal pathfinding was observed at 72.3% of the injection sites after dorsal targeting, compared to 70.6 % after unilateral targeting. Dorsal targeting of an EGFP-expression plasmid did not interfere with axon guidance. All axons had crossed the floor plate by HH25 (**C** and **D**). Bar 100 µm.
