# Supplementary material for: RabGDI controls axonal midline crossing by regulating Robo1 surface expression
Source: Neural Dev. 2012 Nov 9;7:36. doi: 10.1186/1749-8104-7-36 (PMC3520763; doi:10.1186/1749-8104-7-36)
Supplement: Additional file 2 — Figure S2. Downregulation of RabGDI does not interfere with spinal cord patterning. Transverse sections of HH23 embryos were stained with Pax3 (A and B), Nkx2.2 (C and D), and Isl-1 (E and F) to assess spinal cord patterning. The electroporation of dsRabGDI did not change the expression of Pax3 (B), Nkx2.2 (D), or Isl-1 (F). Inserts in B,D, and F show EGFP expression from a co-injected plasmid. A, C, and E show sections taken from a non-injected, age-matched control embryo. [file 1749-8104-7-36-S2.pdf]

Additional Figure 2

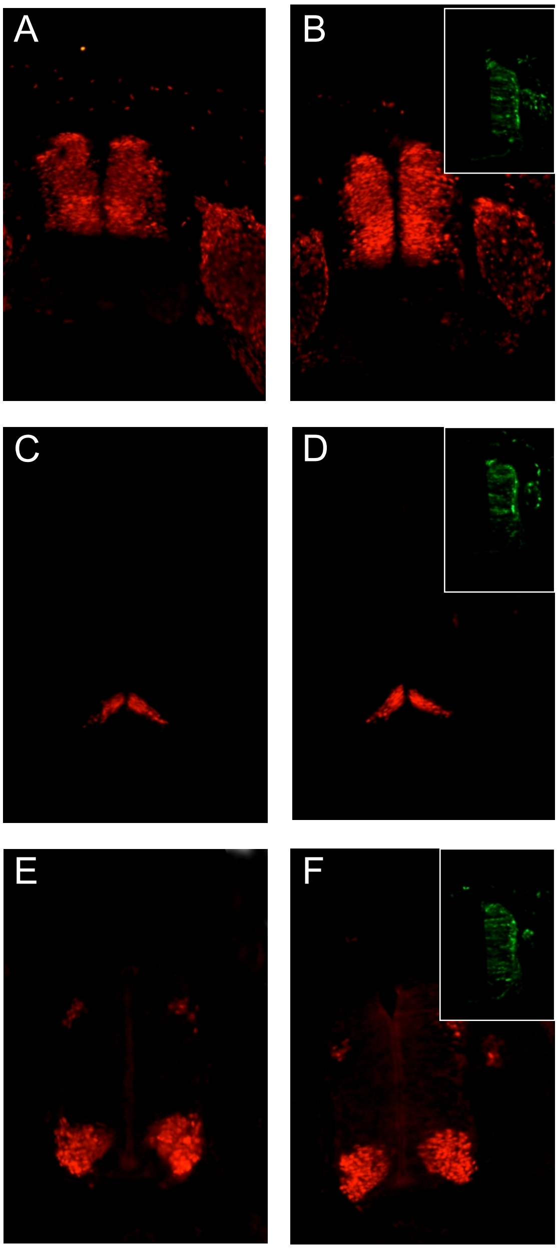

Downregulation of RabGDI does not interfere with spinal cord patterning.

Transverse sections of HH23 embryos were stained with Pax3 (**A** and **B**), Nkx2.2 (**C** and **D**), and Isl-1 (**E** and **F**) to assess spinal cord patterning. The electroporation of dsRabGDI did not change the expression of Pax3 (B), Nkx2.2 (D), or Isl-1 (F). Inserts in B,D, and F show EGFP expression from a co-injected plasmid. A, C, and E show sections taken from a non-injected, age-matched control embryo.
